# Cognitive dysfunctions in high myopia: An overview of potential neural morpho-functional mechanisms

**DOI:** 10.3389/fneur.2022.1022944

**Published:** 2022-11-03

**Authors:** Kaixiu Li, Qun Wang, Liqiang Wang, Yifei Huang

**Affiliations:** ^1^Department of Ophthalmology, The Third Medical Center, Chinese PLA General Hospital, Beijing, China; ^2^Medical School of Chinese PLA, Beijing, China

**Keywords:** Parkinson's disease, cognitive impairment, dementia, high myopia, MRI, neuroimage, the visual system, neuroplasticity

## Abstract

Dementia and cognitive impairment (CIM) carry high levels of mortality. Visual impairment (VI) is linked with CIM risk. High myopia (HM) is a chronic disease frequently leading to irreversible blindness. Current opinion has shifted from retinal injury as the cause of HM to the condition being considered an eye-brain disease. However, the pathogenesis of this disease and the manner in which neural structures are damaged are poorly understood. This review comprehensively discusses the relationship between HM, the central nervous system, and CIM, together with the novel concept of three visual pathways, and possible research perspectives.

## Introduction

The prevalence of cognitive impairment (CIM) is estimated to triple by 2050 (1), as the proportion of the population over the age of 60 is predicted to reach 2 billion people by 2050 (2). CIM is the typical symptom of Alzheimer's dementia. Currently, approximately 6.2 million people in the USA are thought to suffer from Alzheimer's disease (AD) (3), and this could increase to 13.8 million by 2060 without the development of new treatments for the disease (3). AD and other types of dementia are ranked among the top 10 causes of death throughout the world and were ranked third over North and South America and Europe in 2019 by the WHO in 2020.

CIM currently ranks fifth in causes of disability in the elderly (4), leading to often severe challenges, including economic and social as well as psychological and physical burdens to the patients, families, and communities (5, 6). There are limited treatments for dementia (7). Thus, the identification of risk factors that could be addressed in the early stages of the disorder may be more effective than pharmaceutical strategies. This review discusses the role of visual impairment (VI) as a CIM risk factor (8).

Myopia is estimated to affect 28.3% (~2 billion individuals) of the world's population. Of these, 277 million (4.0% of the overall population) suffer from high myopia (HM). HM is the most common cause of blindness in some communities (9, 10). Worse still, HM is expected to increase in prevalence to affect almost 1 billion individuals or 9.8% of the global population in 2050 (11).

Both VI and CIM are relatively common in the elderly and result in significant economic and social burdens. These burdens could be reduced if the disorders could be timeously detected and suitably managed. VI is a documented CIM risk factor. Thus, HM-associated VI may be linked to cognitive decline. A review of the current literature shows that few studies have specifically addressed this issue, with most focusing on the relationships between neural morphology and function, using neuroimaging techniques. Moreover, many of these findings are equivocal. Here, we review the underlying concepts and current published evidence to investigate and understand the association between HM and CIM.

## Association of ophthalmic manifestations with neurogeneration

The brain is specifically linked to the sense organs. In the case of vision, the eye forms an extension of the brain with both organs consisting of neurons derived from the embryonic neural tube. The eye is often described as a “window into the brain” as considerable amounts of vascular and neuroanatomical information on the brain can be collected in a non-invasive manner through the eye. This strong association between the eye and the central nervous system (CNS) is confirmed by the observation that ophthalmic changes occur during neurogenerative disorders of the CNS (12–14). For instance, in AD, β-amyloid plaques are seen not only in the brain but also in the inner retina (15, 16). As it is relatively simple to evaluate the retina using optical techniques such as optical coherence tomography (OCT), specific changes, such as thinning of the retinal ganglion cell layer (GCL) have been detected in early neurodegeneration, reflecting changes occurring in the brain (17). In addition, OCT can identify significant changes in the intraretinal vascular network in AD (18). A variety of ophthalmic problems have been described in patients suffering from neurodegeneration, most commonly AD, including damage to the extraocular muscles, changes in the pupil, and thinning of the RNFL and GCL, as well as visual complaints, such as reduced color and contrast sensitivities, and changes to the visual field (19–26). These issues have also been reported in other neurodegenerative diseases, specifically, Parkinson's disease (PD). PD is accompanied by a wide range of visual problems, involving most of the ocular and visual system components (27, 28), and appear to be identical to the problems described in AD. It thus appears that the eye is not merely a reflection of the neurodegenerative changes occurring within the CNS but may be directly linked with the pathogenesis. In this section, we discuss the eye in the context of injury caused by pathological changes originating within the brain. The question arises whether primary degenerative changes in ocular structures can induce changes in the CNS. Evidence supporting this has shown that neurodegenerative processes involve both the central nervous system and the retina, with anterograde (postsynaptic neurons) and retrograde (presynaptic neurons) transsynaptic neurodegeneration (29). Retrograde transsynaptic neurodegeneration frequently leads to the death of retinal ganglion cells. The following section discusses changes in the CNS resulting from primary disorders of the eye.

## Neurodegenerative manifestations in ophthalmic diseases

The potential participation of the CNS in ophthalmic disease has attracted the attention of both the scientific and clinical communities (30). The optic nerve forms a direct connection between the eye and the brain, and recent evidence has suggested the existence of interrelationships between the two organs on both morphological and functional levels, including the sharing of neurodegenerative features (29, 31). The role of retrograde transsynaptic degeneration has recently been documented in the visual system, specifically in connection with glaucoma (32–34). This mechanism has also been linked to disorders of other posterior segments. Age-related macular degeneration (AMD) leads to degeneration of visual pathways associated with alterations of the connectivity networks in the CNS, resulting in visual impairment (35–37). Genetic retinal dystrophies may show similar features, including alterations in white matter networks and cortical remapping (38–40). Neuronal damage leads to disrupted intercellular communication to the point where entire networks collapse. In transsynaptic degeneration, the damage extends from the primary injury site to distant sites through neuronal communication. Changes in the peripheral visual system initially lead to attempted local compensation, followed at later stages by spreading to the brain accompanied by further impairment (41). Thus, although insufficiently investigated, the interconnections between the eye and the CNS are likely to have profound implications for both understanding visual processing and diseases affecting the two organs.

## VI is a risk factor for CIM

**Figure 1:**
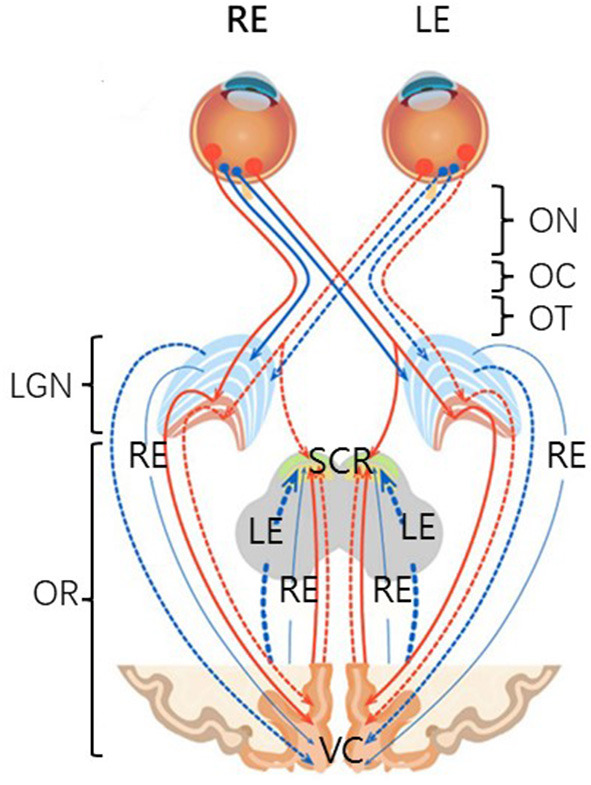
A schematic anatomical overview of the visual system. There are six major subdivisions, including optic nerve (ON), optic chiasm (OC), optic tract (OT), lateral geniculate nucleus of the thalamus (LGN), optic radiation (OR), and visual cortex (VC). Four non-decussating fiber pathways (nasal fibers, blue, and orange) connect the optic nerve and the optic tract in the same hemisphere, while four decussating fiber pathways (lateral fibers, blue, and orange) connect the optic nerve and the optic tract across the hemispheres. SCR, subcortical regions.

In mammals, stimuli entering the eye are processed initially by the retinal mosaic and transmitted to the retinal ganglion cells. The axons of these cells constitute the optic nerve, leading to the lateral geniculate nucleus in the thalamus, after which the signal is transmitted to the primary visual cortex (42, 43) (Figure 1). Thus, retinal damage affects a variety of brain regions and systems, including the subcortical retinofugal system, and the primary, higher, and non-visual cortical areas (44–48). Retinal damage can result in VI, which is a risk factor for CIM (8). Indeed, it has been suggested that VI may be an early symptom of CIM (49).

Neuroimaging investigations have demonstrated changes in the brain not only in neuropsychiatric disorders, such as CIM but also in disorders involving long-term aberrations in sensory input. Specifically, both hearing disorders and amblyopia lead to reductions in the gray matter of the brain. Furthermore, a number of systematic reviews and meta-analyses have suggested that sensory impairment, including VI and hearing and olfactory deficiencies, are risk factors for CIM (8, 50, 51). The brain is known to obtain over 80% of its information concerning the environment through the eyes (52). However, the mechanisms responsible for these risks are largely unknown. In VI, it is possible that the loss of visual information may result in cortical atrophy, leading to reorganizational changes in the brain, which have been reported by neuroimaging studies and pathological evaluations (12, 53, 54). In addition, damage to the visual input may lead to impaired perceptual processing, leading, in turn, to reductions in higher-order cognitive functioning (55). Thus, it is suggested that disruptions in the normal retina-brain connection by neurodegenerative changes in CIM may coincide with VI.

HM is one of the most common causes of retinopathy-induced irreversible VI, especially in older adults (≥50 years) (56). As HM is associated with both hypopsia and fundus lesions, it is essential to explore the structural and functional aberrations in the brain associated with HM.

## Associations between CIM and HM

The link between CIM and myopia is a relatively novel concept and cognitive changes associated with HM are not well-documented (57), with a limited number of studies reporting disparate results. There is a certain amount of epidemiological evidence suggesting a link between myopia and cognitive dysfunction, for example, myopic patients have an almost 2-fold increased likelihood of suffering cognitive dysfunction compared with those with normal vision; this relationship remained significant after adjustment for age, sex, income, education, body mass index, and hours of reading and writing per day, as well as for uncorrected refractive errors or best-corrected visual acuity (58, 59). In contrast, another study found no significant association (60).

Despite correction for visual acuity, the functional performance of HM eyes, including contrast sensitivity (61), acuity of resolution (62), visual attention (63, 64), performance on backward visual masking tasks, and temporal retinal responses assessed by electrophysiological techniques (65), remains abnormal compared with individuals with normal vision.

Mammalian behavior is strongly regulated by the solar cycle, and light produces significant effects on both mood and cognition. In humans, light detection occurs exclusively in the retina. The photoreceptors include the classic rods and cones as well as a subpopulation of retinal ganglion cells that express the photopigment melanopsin (Opn4); these cells are described as intrinsically photosensitive retinal ganglion cells (ipRGCs) (66, 67). The ipRGCs mediate the influence of light through two pathways, namely, targeting the suprachiasmatic nucleus (SCN) in the hypothalamus and the perihabenular nucleus (PHb) in the dorsal thalamus (68). Ablation of the ipRGCs has been shown to completely block light perception in the SCN, indicating that the ipRGCs may be necessary for the modulation of circadian brain functions by light (69). The extended axial length in HM usually results in retinal thinning and retinal thickness has been found to decrease with HM severity. This may also affect the ganglion cells as mediators of mood and cognition.

CIM/Dementia is an overall description that includes a specific group of symptoms associated with deficiencies in memory, language, problem-solving, and cognitive skills that adversely affect the patient's performance in everyday activities. Specific neural regions are involved in cognitive activities, and HM is known to be linked with structural and functional alterations in both the visual and non-visual cortical areas. These overlaps are discussed below.

For example, a report on HM patients (70), observed reductions in the thickness of the cortical surface in the right primary visual area 1 (V1), right occipital pole, and primary motor cortex, amongst others, that were not apparent in healthy controls. Damage to V1 may lead to vision loss while the occipital pole functions during early visual and speech processing, spelling to sound conversion, and modulation of the visual cortex. Another article on HM patients (71) showed white matter abnormalities in the brain regions responsible for motor conduction and vision-related functions. Zhai et al. (72) observed, using fMRI, that HM patients had significantly reduced functional connectivity (FC) between the supramarginal gyrus and rostrolateral prefrontal cortex, together with their associated ventral attention and frontoparietal control networks. These findings indicate the presence of FC alterations in HM and may account for attention deficiency observed in patients with HM. HM is associated with dysfunctional changes in many regions of the brain, possibly indicative of neurobiological alterations associated with the understanding of language and regulation of attention (73). Alterations in the default mode network (DMN), formed by the posterior cingulate cortex, medial prefrontal cortex, and bilateral parietal cortices, have been observed on rsfMRI on patients with CIM and neurodegeneration (74, 75).

Significantly reduced low-frequency fluctuations (ALFFs) in the right inferior and middle temporal gyrus, as well as the left middle temporal gyrus, have been reported in HM patients (73). These regions are proximate to the superior temporal sulcus (STS) and compute a variety of sensory information relating to social interactions (76, 95). The STS is associated with a variety of brain regions linked with the processing of social information (77–82). It has been found that parts of the STS respond to specific visual and auditory stimuli Including facial expression and gaze (83–93), bodies (16, 17, 80, 88), point-light walkers (15, 68, 79, 89, 90), human voices (20, 91), language (21, 92), and the audiovisual integration of speech (22, 93). Furthermore, the temporoparietal junction (TPJ) which lies posterior and superior to the STS controls theory of mind tasks (94), in which participants interpret the actions of characters in stories.

Given the possible relationship between HM and CIM, the recognition of the association between VI from HM and cognitive dysfunction as a complication of HM will allow more successful management of these disorders, reducing economic and healthcare burdens. The use of neuroimaging further confirms these associations.

## Neuroimaging in HM

In this section, the neuroimaging findings are summarized, and we argue that HM-associated changes potentially affect CIM. Abnormal visual experiences are known to influence both the structure and function of the brain. Visual functioning in patients with HM differs significantly from that in individuals with normal vision, despite correction of visual acuity. It is possible that long-term myopic vision may induce alterations in the neural activity of the brain. The most commonly used neuroimaging technique is MRI, which is able to evaluate structure, function, and metabolism throughout the nervous system. However, to the best of our knowledge, there are no reports on metabolic MRI in HM.

In terms of structure, MRI has shown reduced cortical thickness and disconnection in patients with HM (70) where thinning of the cortical surface was seen in the left middle occipital gyrus (MOG), left inferior parietal lobe (IPL), right inferior temporal gyrus (ITG), right precuneus, right primary visual area 1 (V1), right superior temporal gyrus (STG), right superior parietal lobule (SPL), right occipital pole, and right primary motor cortex (M1). Higher white matter concentrations have also been reported in HM patients, particularly in the calcarine area, as well as in smaller areas of the prefrontal and parietal lobes (95). Additional abnormalities in the white matter were observed in the bilateral corticospinal tract, right inferior longitudinal fasciculus, superior longitudinal fasciculus, inferior fronto-occipital fasciculus, and left thalamus (71). A further study using a specific MRI sequence, observed abnormal blood perfusion in the cerebellum of patients with HM (96), providing a different perspective on changes and plasticity in the brains of HM patients.

Brain FC in HM patients has been investigated using fMRI. For instance, Zhai et al. (72) described reduced FC density (FCD) in the posterior cingulate cortex/precuneus of HM patients, together with reduced long-range FCD in the inferior temporal gyrus, supramarginal gyrus, and rostrolateral prefrontal cortex. The reduced FC was seen not only in these regions but also in the networks they belong to, namely, the ventral attention and frontoparietal control networks. These findings indicate changes in FC in HM and may assist in explaining attention deficits often seen in high myopes.

Moreover, at-rest functional MRI (RS-fMRI) using BOLD signals has been successfully used for the investigation of spontaneous neural activity in myopia. One study using this technique (73) observed that patients with HM showed significantly reduced ALFFs in the right inferior and middle temporal gyrus, left middle temporal gyrus, left inferior frontal gyrus/putamen, right inferior frontal gyrus/putamen/insula, right middle frontal gyrus, and right inferior parietal lobule, with raised ALFFs in the bilateral midcingulate cortex, left postcentral gyrus, and left precuneus/inferior parietal lobule. Patients with HM showed multiple abnormalities, which may account for deficiencies in understanding language and the regulation of attention seen in HM patients. A further study compared brain activity between patients with low or moderate myopia (LMM) and those with HM in terms of the ALFF (97), finding differences in activity in the limbic system, default mode network, and thalamo-occipital pathway. Specifically, although abnormalities in the visual and sensorimotor regions of the brain were seen in both LMM and HM patients, those with HM showed greater abnormalities in the border brain areas. In addition, patients with HM showed aberrant activity in both the limbic system and default mode network (DMN). Changes in the DMN which includes the posterior cingulate cortex (PCC), medial prefrontal cortex (mPFC), and bilateral parietal cortices in RS-fMRI have been reported in both CIM and neurodegeneration (74, 75). All the above brain regions, together with the adjacent areas, may be associated with the processing of information concerning cognition-related social interactions.

The question arises as to whether these cortical changes associated with HM are reversible. The answer lies in the plasticity of the adult cerebral cortex. The visual system is considered to be sufficiently plastic to allow modifications of the cerebral cortex only during the first decade of life. However, evidence suggests that the adult visual system may also have some degree of neural plasticity. A specific example concerns the use of refractive surgery for myopia. Abnormal activation of the visual cortex has been reported after corneal refractive surgery for myopia (98); this was observed in all patients with the highest increases in signals seen in the primary visual cortex. Additional signals were observed in the posterior cingulum, anterior cingulum, prefrontal cortex, and frontal cortex. A recent investigation into adult amblyopia observed aberrant FC between the visual cortex and subcortical regions such as the geniculostriate and corticotectal pathways, indicative of the effects of changed visual input on the brain neural activity (43). It has been hypothesized that anisometropic non-amblyopic patients may also suffer relative visual deprivation and that this could be reduced after refractive surgery by plasticity in the visual cortex. This was supported by a study on the effects of refractive surgery in anisometropic adult patients (99) that observed plastic changes in the primary visual cortex. Refractive surgery was found to improve the BSCVA in these patients, counteracting the idea that such visual defects are permanent (100). The control group comprised myopic non-anisometropic subjects. While improvement in visual acuity was observed in both groups, the improvement in the anisometropic patients was slower. As shown by fMRI studies on neuronal, specifically synaptic, activity in relation to increased blood flow, it appears that the visual cortex in adult anisometropic subjects undergoes plastic changes after refractive surgery (101).

## Current limitations and future perspectives

MRI-HM analysis has demonstrated that HM results in changes in both the FC and morphology of the brain cortices. Despite limited epidemiological evidence linking HM and cognitive dysfunction (58–60), relatively little is known of cognitive changes in the presence of HM. To clarify the situation, two limitations of past studies should be discussed.

### Lack of non-invasive neuroimaging with high spatial resolution in HM

The spatial resolution of 1.5 and 3T MRI imaging is relatively low, suggesting that equipment, such as 7T MRI, should be used for higher image resolution in HM investigations. At present, 7T MRI represents the most effective non-invasive technique for neuroimaging. The increased signal-to-noise ratios (SNRs) of 7T compared with conventional 1.5 and 3 T scanners have led to discussions on the benefits of the technology to neuro-ophthalmology (102, 103). Due to the technical limitations in identifying layer-specific activities in the subcortical nuclei in HM, very little is known of the effects of HM on the neuronal responses and circuitry of the subcortical visual pathways.

The initial stages of visual perception are controlled by two parallel pathways (retinogeniculocortical pathways), namely, the magnocellular and parvocellular pathways, which each transmit different types of visual information (43, 45, 104, 105). Physiological and behavioral studies have shown that the magnocellular pathway is most affected in HM, with patients showing impaired perception of motion and blue-yellow contrast (104). To date, there have been no reports on the neuroimaging of the retinogeniculocortical pathways in HM. However, these pathways, together with other subcortical regions, have been investigated in amblyopia using 7 T MRI (43).

### No common framework for investigating the primate visual cortex

There appears to be no consensus on the cortical regions associated with HM from MRI studies. A possible explanation is the lack of a common framework for the investigation of the primate visual cortex.

**Figure 2:**
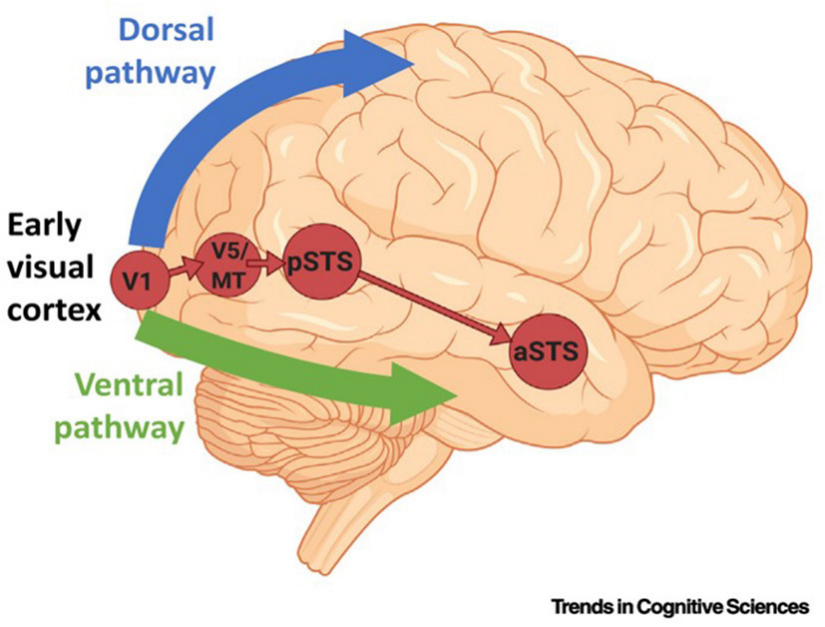
The pathway begins in the primary visual cortex (V1) and projects into the posterior banks of the superior temporal sulcus (STC) *via* the motion-selective area V5/middle temporal (MT). The corticocortical connections of the third pathway are independent of the ventral pathway (in green) and the dorsal pathway (in blue). a/pSTS, anterior/posterior STS.

A recent report by Pitcher, D. et al. described a third visual pathway specifically adapted for social perception [Figure 2, (106)]. These authors proposed the presence of a third visual pathway on the lateral brain surface that is anatomically distinct from the dorsal and ventral pathways. This third pathway is present in both human and non-human primates. In humans, the pathway projects from the early visual cortex into the superior temporal sulcus (STS) (77–82), which has been found to be associated with social cognition. This indicates that the two-visual pathway model of the primate visual cortex requires updating. The ventral pathway is responsible for identifying an object, while the dorsal pathway analyzes the object's location and actions associated with the object. Thus, the two-pathway model is essentially concerned with the “what,” “where,” and “how” of visual object recognition. However, while these attributes can describe many characteristics of visual objects, the terms are wholly inadequate for assessing the complexity and nuances of even basic social interactions. It is apparent that revision to this model is necessary. The presence of the third visual pathway has been documented by tractography investigations in humans and tracer and neuroanatomical studies in non-human primates (106). Various STS subregions have been shown to respond to specific types of social stimuli, such as visual or auditory stimuli, in a posterior-to-anterior direction. It has been reported that the STS is able to compute facial and body movements, such as expression, eye gaze, intention, audio-visual integration, and mood, suggesting specialization of the third pathway for the dynamic aspects of social perception (88, 91, 93, 107, 108). These functions of the STS and, by extension, of the third pathway, are well-documented (88, 107, 108). These findings create a framework for the further study of the cognitive functions of the primate visual cortex. Many experimental methods could be used for this, including neuroimaging (MRI) to evaluate neural responses to abnormal visual stimuli in HM.

## Neural plasticity in adulthood

It is feasible that VI may lead to cognitive decline through the limitation of the individual's social activities, resulting in social isolation and reduced participation in mentally stimulating activities (109, 110). Numerous age-associated eye conditions, such as glaucoma and AMD, that are related to VI have been associated with CIM (111, 112). HM is also documented to be a leading cause of VI. CIM results from neuronal injury and death, together with aberrant synaptic communication. Healthy adult brains contain ~100 billion neurons, each of which connects *via* synapses with other neurons. Synapses allow the rapid transmission of signals within the brain, with the transmitted information forming the cellular basis of cognition.

Brain plasticity may be defined as the capacity of these synaptic connections to adapt in response to stimuli, such as injury, sensory deprivation, and environmental change. Thus, it is possible that changes in the FC or ALFF (shown by MRI) may initiate or promote the progression of neurodegenerative change. AD, which accounts for approximately 60–80% of CIM cases, is progressive and may even start 20 years or more before the appearance of symptoms (113–115). Clinically, the major risk factor for AD is older age (>65 years, although this is not fixed) (116). It has been suggested that degenerative changes in the brain may begin in the fourth decade of life. HM is a major cause of irreversible retinopathy-associated VI, especially in older patients (≥50 years) (56). In addition, HM is a good example of the existence of neural plasticity after development as it is usually of long duration. Plasticity is a distinctive, although not exclusive, feature of the developing nervous system; increasing numbers of experimental studies have indicated that plasticity occurs in the adult human cortex as well (117). It appears that there is a spatial and temporal overlap in the age of onset or progression between HM and CIM.

In terms of the bidirectional association between VI and CIM, Vu, T. A (8) observed using clinically validated cognitive screening tests that VI is a risk factor for CIM, although it is not known if CIM is a risk factor for VI. As both diseases may be present in elderly patients, it is possible that maladaptive alterations in the circuitry of the visual cortices could occur as in neurodegeneration (118). Such changes could result from qualitative or quantitative alterations in neural activity and could be of long duration. As discussed, cortical changes have been demonstrated after corneal refractive surgery, demonstrating the existence of plasticity in the adult cerebral cortex. From this perspective, we, as clinical ophthalmologists, suggest that if HM patients have their refractive surgeries (corneal refraction or posterior chamber phakic intraocular lens) in their third or fourth decades, the chances of their developing CIM later in life will be diminished.

## Conclusion

This review discusses the main findings on the commonalities between neurocognitive structure and function in HM and CIM/dementia. Nevertheless, the interpretation of the association between HM and CIM is both complex and equivocal. We consider that neuroimaging may assist in the exploration of the underlying pathophysiology of CIM and HM. Characterization of the neural plasticity and reorganization resulting from VI is necessary for the development of effective disease management and rehabilitation.

The use of functional neuroimaging techniques with higher resolution, such as 7 T fMRI, would improve this type of analysis. In addition, further investigations into the framework of the three visual pathways, including both tractography studies in humans and tracer and neuroanatomical studies in non-human primates are required for a comprehensive evaluation of the bidirectional relationship between HM and CIM and to investigate the efficacy of vision-saving interventions in preventing the onset and progression of cognitive decline.

## Author contributions

YH and LW designed the study. KL wrote the initial draft. QW, KL, and YH revised the manuscript. All authors listed have made a substantial, direct, and intellectual contribution to the work and approved it for publication.

## Funding

This work was supported by Equipment Comprehensive Scientific Research Project of China (Grant No. LB20201A010024).

## Conflict of interest

The authors declare that the research was conducted in the absence of any commercial or financial relationships that could be construed as a potential conflict of interest.

## Publisher's note

All claims expressed in this article are solely those of the authors and do not necessarily represent those of their affiliated organizations, or those of the publisher, the editors and the reviewers. Any product that may be evaluated in this article, or claim that may be made by its manufacturer, is not guaranteed or endorsed by the publisher.
